# ECG and Biomarker Profile in Patients with Acute Heart Failure: A Pilot Study

**DOI:** 10.3390/diagnostics12123037

**Published:** 2022-12-03

**Authors:** Adriana Chetran, Alexandru Dan Costache, Carmen Iulia Ciongradi, Stefania Teodora Duca, Ovidiu Mitu, Victorita Sorodoc, Corina Maria Cianga, Cristina Tuchilus, Ivona Mitu, Raluca Daria Mitea, Minerva Codruta Badescu, Irina Afrasanie, Bogdan Huzum, Stefana Maria Moisa, Cristian Sorin Prepeliuc, Mihai Roca, Irina Iuliana Costache

**Affiliations:** 1 Department of Internal Medicine, Faculty of Medicine, University of Medicine and Pharmacy “Gr. T. Popa”, 700115 Iasi, Romania; 2 Cardiology Clinic, Clinical Emergency Hospital “Sfantul Spiridon”, 700111 Iasi, Romania; 3 Department of Cardiovascular Rehabilitation, Clinical Rehabilitation Hospital, 700661 Iasi, Romania; 4 2nd Department of Surgery—Pediatric Surgery and Orthopedics, “Grigore T. Popa” University of Medicine and Pharmacy, 700115 Iași, Romania; 5 Pediatric and Orthopaedic Surgery Clinic, “Sfânta Maria” Emergency Children Hospital, 700309 Iași, Romania; 6 II Internal Medicine Clinic, Clinical Emergency Hospital “Sfantul Spiridon”, 700111 Iasi, Romania; 7 Department of Immunology, Faculty of Medicine, University of Medicine and Pharmacy “Gr. T. Popa”, 700115 Iasi, Romania; 8 Immunology Laboratory, Clinical Emergency Hospital “Sfantul Spiridon”, 700111 Iasi, Romania; 9 Department of Microbiology, Faculty of Medicine, “Grigore T. Popa” University of Medicine and Pharmacy, 16 Universitatii Street, 700115 Iasi, Romania; 10 Microbiology Laboratory, Clinical Emergency Hospital “Sfantul Spiridon”, 700111 Iasi, Romania; 11 Department of Morpho-Functional Sciences II, Faculty of Medicine, University of Medicine and Pharmacy “Gr. T. Popa”, 700115 Iasi, Romania; 12 Department of Cardiology, Faculty of Medicine, University of Medicine and Pharmacy “Lucian Blaga, 550169 Sibiu, Romania; 13 Cardiology Clinic, Clinical Emergency Hospital Sibiu, 550245 Sibiu, Romania; 14 III Internal Medicine Clinic, Clinical Emergency Hospital “Sfantul Spiridon”, 700111 Iasi, Romania; 15 Department of Physiology, Faculty of Medicine, “Grigore T. Popa” University of Medicine and Pharmacy, 700115 Iasi, Romania; 16 Department of Orthopaedics and Traumatology, “Sf. Spiridon” Emergency County Hospital, 700111 Iasi, Romania; 17 Department of Pediatrics, Faculty of Medicine, “Grigore T. Popa” University of Medicine and Pharmacy, 700115 Iasi, Romania; 18 “Saint Parascheva”, Infectious Diseases Clinical Universitary Hospital Iasi, 700116 Iasi, Romania

**Keywords:** acute heart failure, ECG, biomarker, Holter monitoring, NT-proBNP, troponin, cQTi, spot urinary sodium, prognosis

## Abstract

Background: Biomarkers, electrocardiogram (ECG) and Holter ECG are basic, accessible and feasible cardiac investigations. The combination of their results may lead to a more complex predictive model that may improve the clinical approach in acute heart failure (AHF). The main objective was to investigate which ECG parameters are correlated with the usual cardiac biomarkers (prohormone N-terminal proBNP, high-sensitive cardiac troponin I) in patients with acute heart failure, in a population from Romania. The relationship between certain ECG parameters and cardiac biomarkers may support future research on their combined prognostic value. Methods: In this prospective case-control study were included 49 patients with acute heart failure and 31 participants in the control group. For all patients we measured levels of prohormone N-terminal proBNP (NT-proBNP), high-sensitive cardiac troponin I (hs-cTnI) and MB isoenzyme of creatine phosphokinase (CK-MB) and evaluated the 12-lead ECG and 24 h Holter monitoring. Complete clinical and paraclinical evaluation was performed. Results: NT-proBNP level was significantly higher in patients with AHF (*p* < 0.001). In patients with AHF, NT-proBNP correlated with cQTi (*p* = 0.027), pathological Q wave (*p* = 0.029), complex premature ventricular contractions (PVCs) (*p* = 0.034) and ventricular tachycardia (*p* = 0.048). Hs-cTnI and CK-MB were correlated with ST-segment modification (*p* = 0.038; *p* = 0.018) and hs-cTnI alone with complex PVCs (*p* = 0.031). Conclusions: The statistical relationships found between cardiac biomarkers and ECG patterns support the added value of ECG in the diagnosis of AHF. We emphasize the importance of proper ECG analysis of more subtle parameters that can easily be missed. As a non-invasive technique, ECG can be used in the outpatient setting as a warning signal, announcing the acute decompensation of HF. In addition, the information provided by the ECG complements the biomarker results, supporting the diagnosis of AHF in cases of dyspnea of uncertain etiology. Further studies are needed to confirm long-term prognosis in a multi-marker approach.

## 1. Introduction

Heart failure (HF) continues to be one of the leading causes of morbidity and mortality worldwide, as it represents the end stage of most heart diseases. Cases of acute decompensated HF (AHF) are particularly important, since they require a considerable amount of resources for hospitalization and treatment. Since the symptoms are not specific and can interfere with other diseases, diagnosis is often uncertain, delaying appropriate therapeutic intervention [1]. The standard and guideline-recommended approach to diagnosing ACF includes obtaining and interpreting an electrocardiogram (ECG), echocardiography (Echo), and measurement of plasma natriuretic peptide (NP) levels. Additional diagnostic tests such as pulse oximetry, chest X-rays, lung ultrasounds, computed tomography, coronary angiography and Holter ECG monitoring can be helpful to establish an AHF diagnosis, etiology or prognosis [2].

HF is defined as a complex clinical syndrome caused by decreased cardiac output (CO), lack of efficient venous return and inadequate fulfillment of metabolic and tissue demands. HF develops as a secondary cardiac complication of several cardiovascular conditions. Different etiologies alter cardiac function through several mechanisms: pressure overload (arterial hypertension, valvular stenosis), volume overload (valvular regurgitation, intracardiac fistulas, arteriovenous fistulas), decreased contractile efficiency (myocardial ischemia, dilated cardiomyopathies, myocarditis, infiltrative diseases, neuromuscular diseases, endocrine diseases) and decreased cardiac filling (pericardial diseases, intracardiac obstructions, restrictive cardiomyopathies). All of these conditions can lead to left ventricular systolic or diastolic dysfunction. The type of left ventricular dysfunction is determined by the ejection fraction (EF), which quantifies the amount of blood pumped by the ventricle in one heartbeat. Systolic dysfunction is defined by an EF < 40%, while an EF > 40% is characteristic of diastolic dysfunction [3,4]. The current ESC (European Society of Cardiology) guideline for HF recommends using EF-based classification, as an indicator of the underlying mechanism: HF with reduced EF (EF ≤ 40%), HF with mildly reduced EF (EF between 41% and 49%) and HF with preserved EF (EF ≥ 50%) [2].

Decreased CO and insufficient tissue perfusion in HF, trigger several compensatory mechanisms. In the early stages of HF, the Frank–Starling mechanism acts by improving CO through the increasing of LV end-diastolic volume. On the other hand, decreased CO is associated with low blood pressure, which in turn activates the sympathetic nervous system (SNS) and renin–angiotensin–aldosterone system (RAAS). Stimulation of the SNS and release of catecholamines (norepinephrine and epinephrine) have direct effects on the heart (increased heart rate and contractility) and peripheral vasculature (vasoconstriction). Excess catecholamines may play a role in arrhythmias and heart rate variability. RAAS is activated by decreased renal perfusion, leading to sodium–water retention and vasoconstriction. Natriuretic peptides (NPs) are released in response to atrial and ventricular overload and have a role in sodium and water homeostasis, intervening in the regulation of vascular hemodynamics [3,4].

The response of the myocardium to the chronic increase in parietal stress is remodeling, an adaptive process, through which mechanical, neurohumoral and possibly genetic factors affect the size, shape and function of the left ventricle. These dynamic phenomena, starting with myocyte hypertrophy, followed by fibrosis and deposition of extracellular matrix, ending with myocyte apoptosis and necrosis, lead to changes in the geometry of the cavity, with the aim of maintaining the stroke volume, subsequently perpetuating a vicious circle, the process becoming maladaptive [3,4]. Giulia Ottaviani et al. showed in their research on explanted hearts that end-stage heart failure is associated with high degrees of fibrosis, hypertrophy and myocytolysis [5].

The resting electrocardiogram (ECG) is part of the initial evaluation of patients with heart failure according to the guidelines [2]. Its significance is that, while being non-invasive and easy to perform, it can detect a wide range of abnormalities that can be involved in the etiology and severity of HF [6,7]. Ambulatory 24 h Holter ECG monitoring brings complementary information in patients with HF, as it allows the identification of masked arrhythmias, changes in heart rate (heart rate variability) and ventricular repolarization behavior. Myocardial vulnerability assessed by Holter monitoring was associated with an independent risk of total mortality and HF progression [8].

Specific ECG parameters were identified as markers of acute decompensation or prognosis in previous studies. QT prolongation proved to be an independent predictor of total mortality in patients with HF, reduced EF and myocardial infarction [9,10,11]. QT variability, measured on twenty-four-hour digital Holter recordings, predicts spontaneous ventricular arrhythmias and is a marker of an increased risk of all-cause and CV mortality [12,13,14,15]. Fragmented QRS was associated with lower EF in patients with chronic HF, HF progression, diastolic cardiac dysfunction and adverse cardiac events [16,17,18,19]. Moreover, heart-rate turbulence and T-wave alternations have shown predictive ability for hospitalization and cardiac death in patients with HF [20,21,22,23].

The inherent complexity of ECGs and the subtle differences in the ECG waveform have led to abundant research in recent years into new advanced ECG technologies and digital ECG markers. The role of ECG in the diagnosis of various pathologies is strengthened by the results of new automatic analytical systems. Iñigo Monedero showed in its paper that, with the aid of an automatic system, the standard 12-lead ECG may provide the diagnosis for 13 different diseases [24]. Long short-term memory neural networks (LSTM) were used to classify ECG signals by combining LSTM and angle transform (AT) methods, a new approach for the automatic identification of congestive heart failure (CHF) and arrhythmia (ARR) [25]. Furthermore, the ECG diagnostic performance was improved after the categorization of ECG signals based on downsampling one-dimensional-local binary pattern (1D-DS-LBP) and long short-term memory (LSTM) [26]. Since ECG data is considered unique to each person, new evidence shows that ECG signals can be used for personal identification as part of biometric systems [27]. Necati Demir et al. designed a method using one-dimensional multi-layer co-occurrence matrices (1D-MLGLCM) to recognize individuals based on their ECG signal, achieving very efficient results [28]. All of these studies highlight the vast potential of ECG as a diagnostic and prognostic tool in clinical practice. As the evidence in favor of high-tech analysis methods accumulates, the wide availability of manual ECG interpretation supports its role in daily practice. From this perspective, standard ECG analysis remains a cornerstone of HF diagnosis.

A wide variety of portable ECG devices are becoming available to the general population, bringing the ECG to the center of patient care. A review of their use in the clinical setting concluded that the information provided by these ECG devices often resulted in reduced time to diagnosis and lower rates of ED visits [29].

NPs are a family of neurohormones with similar primary amino acid structure but different physiological effects. Currently, three NPs have been identified: atrial natriuretic peptide, also known as ANP; brain natriuretic peptide, also known as BNP (B-type natriuretic peptide); and C-type natriuretic peptide, also known as CNP [30]. ANP is secreted by cardiac muscle cells in the atria, in response to the increased stretch of the atrial wall, due to increased atrial blood volume. ANP acts by increasing renal sodium excretion, leading to a reduction in expanded extracellular fluid (ECF) volume [30]. BNP is secreted by ventricular muscle cells in response to wall stretch, increased ventricular blood volume, neurohormonal activation and hypoxia. Physiological effects of BNP include natriuresis, decrease of systemic vascular resistance and central venous pressure, inhibition of cardiac remodeling and fibrosis processes [30,31]. Once secreted, the propeptide (proBNP) is split into two parts: the biologically active BNP and prohormone N-terminal proBNP (NT-proBNP) [30]. Both ANP and BNP are found in high concentrations in patients with heart failure, attempting to restore extracellular fluid volume homeostasis and counteract pathological effects on cardiomyocytes. However, BNP and NT-proBNP have a biological half-life twice as long as that of ANP, resulting in higher plasma concentrations. Due to these characteristics, BNP and NT-proBNP are considered superior to ANP for heart failure diagnosis [32,33]. CNP is a structurally related molecule to the other NPs, involved in the simulation of long bone growth, without having natriuretic activity. However, certain studies suggest that CNP may reduce cardiac remodeling in experimentally induced myocardial infarction [30,34]. CNP has a low concentration in normal human subjects, which may be elevated in patients with congestive heart failure but to a much lesser extent compared to ANP and BNP [30,35].

BNP and NT-proBNP are the current established biomarkers for confirming the diagnosis of AHF, assessing the prognosis and monitoring disease progression [6]. The diagnosis of AHF may be excluded if NP concentrations are normal, while the rule-in values for the diagnosis of AHF are: BNP > 100 ng/L and NT-proBNP > 300 ng/L. The values for NT-proBNP should be adjusted by age, as age proved to be a physiological factor that influences NP, so the cutt-off for rule-in AHF are: NT-proBNP > 450 ng/L if aged < 55 years, >900 ng/L if aged between 55 and 75 years and >1800 ng/L if aged > 75 years [2,36]. However, the NPs are not specific for AHF, so the interpretation should take into consideration all of the cardiac or non-cardiac conditions that may elevate NPs levels: atrial fibrillation, cardioversion, myocarditis, hypertrophic or restrictive cardiomyopathy, pulmonary hypertension, pulmonary embolism, acute coronary syndrome, ischemic stroke, renal dysfunction, chronic obstructive disease, severe infections (pneumonia or sepsis), anemia, severe metabolic and hormone abnormalities (thyrotoxicosis, diabetic ketosis), severe burns [37].

Troponins (cTn) are cardiac proteins, found in the cytoplasm of cardiac myocytes, involved in the cardiac muscle contraction by facilitating the interaction between actin and myosin filaments. Cardiac troponins are divided in 3 subunits: cTnC, cTnI and cTnT. cTnI and cTnT have greater sensitivity and specificity for cardiac muscle compared to cTnC, which established them as biomarkers of cardiac muscle injury. However, since small amounts of cTnT have been found in skeletal muscle, cTnI is considered to be the most specific subunit for cardiac muscle [38,39]. As a myocardial infarction occurs and myocytes cell membranes are ruptured, troponins are released in the bloodstream, which leads to detectable levels in the blood. The discovery of this mechanism led to the use of troponin levels in the diagnosis of myocardial infarction [38,40,41].

Elevated troponin levels were also observed in the absence of acute myocardial infarction, suggesting the involvement of multiple mechanisms, including necrosis, apoptosis, necroptosis, cellular injury and decreased clearance [42]. Recently developed improved cTnI and cTnT detection methods, called highly sensitive methods (hs-cTnI, hs-cTnT), have changed the perspective on the biology and diagnostic capability of these biomarkers [39,43]. Cardiac and non-cardiac pathologies may be associated with high troponin levels, through cardiomyocytes injury by different mechanism: cardiomyopathies, heart failure, myocarditis, cardiotoxic drugs, takotsubo syndrome, heart surgery, pulmonary embolism, sepsis, chronic renal disease, chronic obstructive pulmonary disease, systemic hypoxia, severe anemia [39].

The MB isoenzyme of creatine phosphokinase (CK-MB) is an enzyme present in the cardiac muscle, and it participates in the conversion of ATP to ADP. As an indicator of cardiac muscle damage, with a rapid increase in circulating levels, CK-MB is mainly used in the diagnosis of myocardial infarction [38]. In patients with heart failure, CK-MB has shown its utility as a prognostic biomarker, higher serum concentrations being correlated with the clinical severity of the disease [44].

The role of ECG in the diagnosis and prognosis in acute heart failure is well established by previous data. Cardiac biomarkers are essential for establishing the diagnosis, bringing valuable information about the short and long-term prognosis. The relationship between cardiac biomarkers and specific ECG parameters in patients with acute HF has not been previously investigated. Furthermore, it is well known that differences in ethnic and cultural origins among enrolled populations can significantly influence the risk evaluation in patients with heart failure [45]. Therefore, we considered it necessary to explore and report this data from Romania. The aim of this study was to identify which ECG parameters are correlated with biomarker levels in patients with acute heart failure, in an adult population from Romania. The results may improve ECG interpretation in patients with acute dyspnea and provide data for future research on a more complex risk stratification model.

## 2. Materials and Methods

### 2.1. Study Design and Population

We conducted a prospective case-control study on consecutive adult patients with acute onset dyspnea due to acute heart failure, admitted to our emergency hospital (Saint Spiridon County Hospital Iaşi Romania) from February 2022 to September 2022. The patients were followed up on during hospitalization in the Cardiology Department. For the control group, we included ambulatory patients, without HF or with stable compensated HF. The exclusion criteria were: all patients aged < 18 years, presence of pregnancy, value of NT-proBNP < 300 pg/mL, patients with end-stage malignancies.

A fasting venous blood sample was collected from all patients within 72 h after hospitalization, in order to determine the levels of cardiac biomarkers: NT-proBNP, hs-cTnI and CK-MB. Among natriuretic peptides, we measured plasma NT-proBNP levels, as it proved to have higher blood concentrations compared to BNP and ANP in heart failure [2,33]. Among cardiac troponins, we evaluated high-sensitivity cardiac troponin I (hs-cTnI), as recommended by International Guidelines [46]. The biomarkers were measured using PATHFAST Cardiac Biomarker Analyzer (LSI Medience Corporation, Tokyo, Japan), Sysmex XT-4000i—Automated Hematology Analyzer (Sysmex Corporation, Tokyo, Japan) and ARCHITECT c16000 Clinical Chemistry Analyzer (Abbott Laboratories, Abbot Park, IL, USA). The PATHFAST NT-proBNP and hs-cTnI assay principle is based on the chemiluminiscence enzyme immune assay and *MAGTRATION^®^ methodology, and the manufacturer reference interval is <15–128 pg/mL for the NT-proBNP and 0–29 ng/L for hs-cTnI, with the 99th percentile of URL of 29.7 ng/L for males and 20.3 ng/L for females.

The laboratory parameters were completed with standard blood tests: D-dimers, blood count, renal function, liver function, C reactive protein, spot urinary sodium concentration (UNa), urine albumin to creatinine ratio (ACR), microalbuminuria, spot urinary creatinine concentration, uric acid, total protein, thyroid function, serum iron, ferritin, serum lactate.

A standard 12-lead ECG, two-dimensional echocardiography and 24-h Holter ECG monitorization were performed in all included subjects. The parameters analyzed on the 12-lead ECG were: PR/corrected QT (QTc)/QRS intervals, rhythm type (sinus rhythm/atrial fibrillation/atrial flutter/pacemaker rhythm/paroxysmal supraventricular tachycardia), heart rate, the presence of Q wave/U wave/negative T wave/changes of the ST segment/fragmented QRS (fQRS)/poor r-wave progression (PRWP)/low QRS voltage (LQRSV)/extrasystoles/intraventricular or atrioventricular conduction disturbance/left ventricular hypertrophy.

We recorded 24 h Holter ECG for 62 patients (77.5%) using a Cardiospy Holter machine. The parameters analyzed on the Holter ECG tracings were: standard deviation of the NN (R-R) intervals (SDNN), standard deviation of the average normal-to-normal (NN) intervals (SDANN), root mean square of the successive differences (RMSSD), HRVTi (heart rate variability triangular index), the presence of extrasystoles, atrial fibrillation, atrial flutter, ventricular tachycardia. The ECG readings were manually inspected, and all of the intervals containing artifacts and ectopic beats were excluded, replaced by the preceding normal RR intervals. Patients with no sinus rhythm were excluded from the analysis of HRV.

Echocardiography was performed for all patients, using a GE Vivid S70 echocardiography device.

### 2.2. Definitions

Acute heart failure was considered for patients that met the Framingham criteria. The major criteria are: acute pulmonary edema on X-ray, cardiomegaly (cardiothoracic index > 0.5 on X-ray), paroxysmal nocturnal dyspnea, orthopnea, jugular vein distension, hepatojugular reflux, pulmonary rales, the presence of the third heart sound (gallop rhythm), weight loss > 4.5 kg in 5 days in response to treatment. The minor criteria are: ankle edema, dyspnea on exertion, hepatomegaly, nocturnal cough, pleural effusion, tachycardia (heart rate > 120/min). For the diagnosis, at least 2 major criteria or 1 major criterion plus 2 minor criteria are required.

The ECG parameters were defined as follows:

- left ventricular hypertrophy (LVH) was considered in patients that met the Estes criteria and Morris index (point score ≥ 5);
- correction of the QT interval (QTc) was done using the Fridericia formula;
- a fragmented QRS complex (fQRS) was defined by: the presence of notched R or S or the existence of an additional wave-like RSR’ pattern in the original QRS complex, a duration of <120 ms, not accompanied by a typical bundle branch block;
- poor R-wave progression (PRWP) was considered after exclusion of LVH features: RV3 or RV4 < 2 mm plus a decrease in RV2 to RV3 or RV3 to RV4, RV3 < 1 mm plus < 0.25 mm increase from RV2 to RV3;
- low QRS voltage (LQRSV) was defined as a peak-to-peak QRS amplitude of <5 mm in the limb leads and/or <10 mm in the precordial leads;
- left bundle branch block (LBBB) was defined by: QRS duration > 120 ms, dominant S wave in V1, broad monophasic R wave in lateral leads (DI, aVL, V5, V6), absence of Q wave in lateral leads, prolonged R wave peak time > 60 ms in V5–V6;
- pathological Q wave was considered if: >40 ms wide (>1 mm), >2 mm deep, >25% of depth of QRS complex, seen in leads V1–V3;
- complex premature ventricular contractions (PVC) were considered in the presence of: doublets, triplets or non-sustained ventricular tachycardia (NSVT).
- For cardiac biomarkers the normal range was defined according to the following cut-off values: NT-proBNP < 300 pg/mL, high-sensitive cardiac troponin < 14 ng/L, CK-MB ≤ 16 U/L, D-dimers < 500 µg/L.

### 2.3. Statistical Analysis

For statistical analysis, we used IBM SPSS statistical software, version 19.0 (SPSS Inc., Chicago, IL, USA). The categorical variables were expressed as frequencies (numbers) and percentages (%). For the quantitative variables we performed the Shapiro–Wilk test for the assessment of the variables’ normal distribution within the study population. The deviations from normality was considered for *p* value < 0.05 with a Shapiro–Wilk test. Normally distributed variables were expressed as means ± standard deviation (STD), while non-normally distributed data were presented as medians with interquartile ranges (IQRs: 25–75%). For biomarker values that were not normally distributed, we performed log-transformation.

For the comparison of parametric values between the two groups we used means of two independent sample Student’s t or Mann–Whitney U tests. The comparison of categorical variables was performed using the chi-square or Fisher’s exact test. Assessment of the correlations between different parameters was performed using Pearson’s (for continuous variables) or Spearman’s (for categorical variables) coefficients (r). Statistical significance was considered when a two-sided *p*-value was <0.05.

## 3. Results

### 3.1. Baseline Characteristics

The study included a total of 80 participants. The subjects were divided into two groups: 49 patients with acute decompensated heart failure (AHF) and 31 participants in the control group, representing patients without heart failure or with chronic heart failure. A total of 51 participants were males (63.8%), while 29 were females (36.6%), and the mean age was 65.18 ± 12.98 years (variations between 35 and 88 years). The area of residence was equally represented at 50% for both urban and rural areas (Table 1).

We assessed common cardiovascular risk factors in comparison for the two groups, concluding that they had a similar cardiovascular risk profile, as no statistical difference was observed in acute HF patients versus control group (Table 1).

Comorbidities are frequently associated with heart failure, contributing to the prognosis of the disease. In the group of acute HF patients, we identified a higher prevalence of chronic kidney disease (CKD) (85.7% vs. 66.7%, *p* = 0.046), atrial fibrillation (53.1% vs. 25.8%, *p* = 0.016) and chronic obstructive pulmonary disease (COPD) (24.5% vs. 6.5%, *p* = 0.036).

The mortality rate was 6.5%, all the fatality cases being recorded in patients with acute HF. The absence of statistical significance for mortality was due to the small number of cases. Regarding self-care capacity, there was a significant number of impaired patients in the AHF group compared to the control group (32.7% vs. 6.5%, *p* = 0.006).

As there are specific treatment indications for acute heart failure, we looked at the differences in therapy in the two groups, detailed in Table 2. We identified a higher use of loop-diuretics (95.9% vs. 48.4%, *p* < 0.001), amiodarone (32.7% vs. 9.7%, *p* = 0.019), nitroglycerine (34.7% vs. 0.0%, *p* = 0.001) and CPAP (continuous positive airway pressure) non-invasive ventilation (26.5% vs. 0.0%, *p* = 0.002) in the AHF group.

Meanwhile, beta-blockers, mineralocorticoid receptor antagonists (MRA), inhibitors of the renin–angiotensin–aldosterone system, calcium-channel blockers and sodium-glucose cotransporter-2 (SGLT2) inhibitors were equally prescribed in AHF group and control group, given the fact that they are also indicated in chronic heart failure.

We expected to find a significant difference for dobutamine, vasopressor drugs and invasive ventilation given the greater severity of AHF, but, due to the small number of cases, the hypothesis was not confirmed.

Routine laboratory tests were included in the analysis, with significant higher values detected in acute HF for: leukocytes, electrolytes (calcium, magnesium), renal function (urea, creatinine, urinary sodium (UNa), urinary albumin-to-creatinine ratio (ACR), microalbuminuria (MAU), spot urine creatinine), uric acid, serum iron, markers of cholestasis (total bilirubin, gamma-glutamyl transferase, alkaline phosphatase), lactate dehydrogenase (LDH) and total protein. The detailed values are presented in Table 3.

The detailed characteristics of patients with acute HF are presented in Table 4. For patients with acute HF, the predominant etiology was ischemic (51.1%); the majority of patients had a dyspnea of NYHA class III (58.3%) and had reduced ejection fraction (EF) (81.3%).

Comparing the echocardiographic profiles between the groups, we observed that the severity of parameter changes was significantly associated with acute HF (Table 5). Ejection fraction (EF) was lower in AHF group (30 ± 15.87 vs. 42.96 ± 17.56, *p* = 0.01); the left ventricle was more dilated (58.28 ± 7.87 vs. 54.19 ± 8.19, *p* = 0.029), and the systolic pulmonary artery pressure was higher (45.14 ± 22.51 vs. 29.31 ± 19.64, *p* = 0.002). Besides, as atrial fibrillation is more frequently present in cases with AHF, we expected left atrial dilation to be associated with this group, a theory confirmed by our study (30.14 ± 12.16 vs. 23.78 ± 5.75, *p* = 0.008).

### 3.2. Cardiac Biomarkers Profile in AHF and Control Group

The descriptive statistics for cardiac biomarkers values (NT-proBNP and hs-TnI) are presented in Table 6. The two biomarkers show asymmetric distribution (high skeweness values) and significant deviations from normality (Shapiro–Wilk tests with *p* value < 0.05). The descriptive analysis of log-transformed biomarkers values showed asymmetry of the distribution and significant deviations from normality (Shapiro–Wilk tests with *p* value < 0.05) (Table 7). As the biomarker values had deviations from normality, we compared the biomarker profile between groups, using the Mann–Whitney test. Both NT-proBNP and hs-TnI have significantly higher values in patients with acute HF compared to controls (*p* < 0.001).

### 3.3. ECG and Holter ECG Parameters in AHF and Control Group

The detailed ECG and Holter monitoring data are presented in groups determined by the presence or absence of acute HF in Table 8 and Table 9. The parameters were similar between the groups for heart rate, QRS interval (QRSi), PR interval (PRi), QT interval (QTi). The AHF group had a significant higher cQTi (432.49 ± 79.32, *p* = 0.050) than control group.

In Table 8, we illustrate the frequency of pathological changes in ECG or Holter for the two groups. Abnormal ECG (defined as the presence of any pathological modification) was significantly more present in patients with acute HF (*p* = 0.021), who had a very high percentage (81.6%) of ECG changes. Looking at specific ECG abnormalities, patients with acute HF more frequently had a non-sinus rhythm (*p* = 0.026), prominent U wave (*p* = 0.043), poor R wave progression (PRWP) (*p* = 0.012) and atrial fibrillation (*p* = 0.029). No difference was noted between the groups for pathological Q wave, negative T wave, LQRSV, fQRS, ST-segment modification and complex premature ventricular contractions (PVCs).

Holter ECG monitoring allows analysis of the heart-rate variability through parameters such as SDNN, SDANN, RMDDS and HRVTi, which can be seen in Table 10. In our study, we did not find significant modifications of heart-rate variability parameters in patients with AHF compared to control group.

### 3.4. Correlations of Cardiac Biomarkers with ECG and Holter Parameters

**Figure 1:**
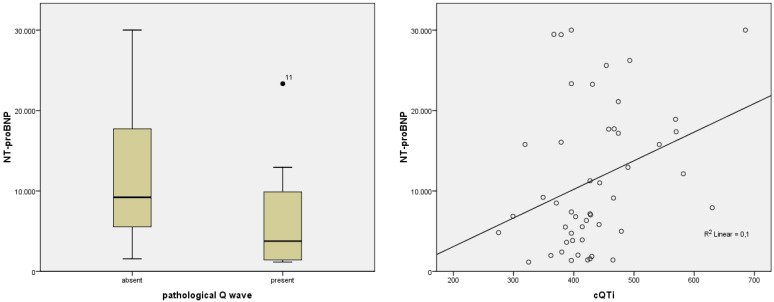
Correlations of NT-proBNP with cQTi and pathological Q wave in patients with AHF. Abbreviations: NT-proBNP = N-terminal (NT)-pro B-type natriuretic peptide; cQTi = corrected QT interval.

In patients with acute HF, we investigated the correlations between different cardiac biomarkers and ECG or Holter parameters. Detailed results are presented in Table 11 and Table 12. For NT-proBNP, we observed a positive significant correlation only with QTi (*p* = 0.039, r = 0.296) and cQTi (*p* = 0.027, r = 0.317), as shown in Figure 1. No correlations were present between parameters of HR variability and cardiac biomarkers (NT-proBNP, hs-cTnI, CK-MB).

**Figure 2:**
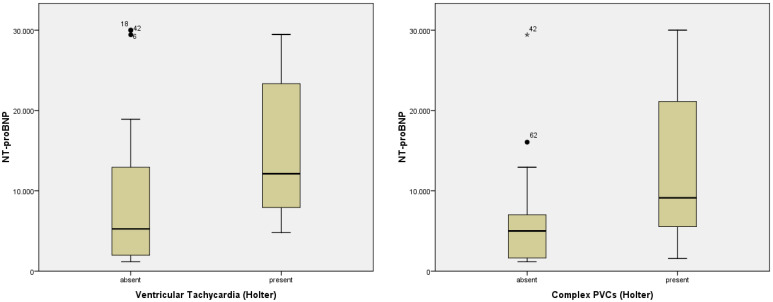
Correlations of NT-proBNP with ventricular tachycardia and complex PVCs in patients with AHF. Abbreviations: NT-proBNP = N-terminal (NT)-pro B-type natriuretic peptide; PVCs = premature ventricular contractions.

Regarding the association of biomarkers with different pathological patterns on ECG and Holter, we found statistical significance for pathological Q wave (*p* = 0.029, F = 5.057), complex PVCs (*p* = 0.034, F = 4.857), ventricular tachycardia (*p* = 0.048; F = 4.167) and NT-proBNP (Figure 1 and Figure 2). hs-cTnI and CK-MB were associated, as expected, with the presence of ST-segment modification. Furthermore, hs-cTnI was able to predict the presence of complex PVCs (*p* = 0.031; F = 4.987).

**Figure 3:**
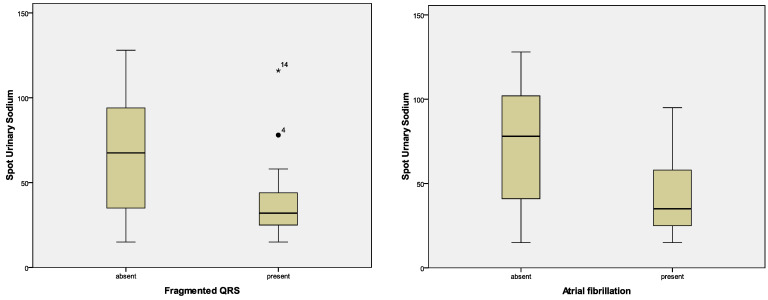
Correlations of spot urinary sodium with fragmented QRS and atrial fibrillation in patients with AHF.

**Figure 4:**
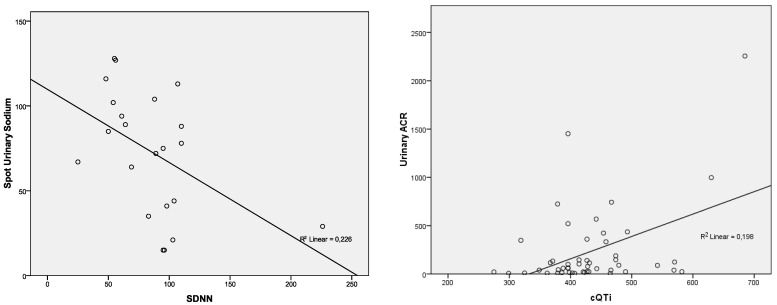
Correlations of spot urinary sodium and SDNN. Correlations of urinary ACR and cQTi in patients with AHF. Abbreviations: SDNN = standard deviation of the NN intervals; cQTi = corrected QT interval; ACR = albumin to creatinine ratio.

Given the significantly modified values of spot UNa and urinary ACR in patients with AHF compared to controls, we investigated possible correlations with ECG parameters and Holter patterns. For spot UNa, we found lower values and strong association with fragmented QRS, atrial fibrillation and SDNN. Urinary ACR showed correlations only with cQTi. Detailed data are in Table 11 and Table 12. Figure 3 and Figure 4 show graphs of the relationship between these parameters.

## 4. Discussion

Acute decompensated heart failure represents one of the most challenging cardiac emergencies. The wide variability of clinical profiles and the severity of underlying cardiac pathology, associated comorbidities, systemic impact and patient vulnerability lead to a wide range of potential complications that impact prognosis. The prognosis of AHF is still poor, despite therapeutic advances, with an in-hospital mortality rate of 4–7%, a 60- to 90-day mortality of 7% to 11% and a 60- to 90-day rehospitalization ranging from 25% to 30%, according to AHF registries [47]. The in-hospital mortality rate in our study was 10.2% for patients with AHF, even higher than that described in other studies, highlighting the risk of AHF and the need for a better understanding of the disease.

When we analyzed the impaired self-care capacity, we found that, for AHF, the percentages were significantly higher compared to the control group, with a rate of 32.7%. This poses additional problems for patients with AHF, who often have an impaired quality of life. The TRIUMPH study (TRanslational Initiative on Unique and novel strategies for Management of Patients with Heart failure) showed that NT-proBNP and comorbidities (COPD, anxiety, depression, HTN) are the main determinants of the quality of life in patients with AHF [48]. According to several HF registries, biomarkers (NT-proBNP, troponins, serum sodium, serum creatinine) and comorbidities (COPD, CKD, diabetes, anemia) are among the predictors of rehospitalization for AHF [47]. In our study, CKD and COPD were the main non-cardiac comorbidities present in patients with AHF, with significantly higher rates than controls (85.7% vs. 66.7% and 24.5% vs. 6.5%, respectively).

Laboratory test results were also comparable to previous studies, as serum iron deficiency, elevated serum creatinine and low urinary sodium levels were more common in patients with AHF. In addition, our analysis showed elevated values for functional liver tests (total bilirubin, GGT, ALP, LDH, total serum proteins) in AHF group compared to controls. Secondary to congestive hepatopathy (CH) or acute cardiogenic liver injury (ACLI), laboratory signs of liver damage have been previously described and have been associated with a higher risk of cardiovascular events and mortality [49,50,51].

Apart from urea and creatinine, we looked for additional, more sensitive markers of kidney damage. Among them, urinary ACR, microalbuminuria (MAU) and spot urine creatinine were found to have abnormal values in patients with AHF, with statistical significance compared to control group. As other studies show, albuminuria is more common in adults with chronic HF (CHF) than in those without CHF; it has clinical value in improving the accuracy of diagnosis of HFpEF (heart failure with preserved ejection fraction) and is a predictor of mortality in patients with HF [52,53,54,55]. In particular, Liang W. et al. showed, in a meta-analysis published in 2021, that MAU was associated with significantly increased cardiovascular mortality and hospitalization [54]. From this perspective, we believe that the assessment of albuminuria should be part of the biomarker profile in patients with HF.

Spot urinary sodium (UNa) provides useful information concerning the volume status, lower concentrations typically reflecting enhanced renal sodium retention in the context of extracellular fluid volume deficit [56]. As Martens et al. observed, lower UNa concentrations were associated with AHF, and a dynamic decrease in UNa concentration was present in the week preceding hospitalization, concluding that UNa sample collection is feasible for detection of AHF and may offer additional prognostic and therapeutic information [57]. During hospitalization, a low UNa characterizes an insufficient natriuretic response to diuretic therapy, with subsequent inadequate decongestion, worsening renal function and adverse long-term events [58,59]. The prognostic value is supported by the correlation with the risk for prolonged hospitalization and all-cause mortality, as several studies suggested [60,61]. For our patients with AHF, UNa had significantly lower levels compared to control group, which supports its power to discriminate decompensated HF from stable HF. As all the previous data showed, UNa has a great potential to become one of the routine biomarkers in AHF.

For our study, the profile of patients with acute heart failure included most frequently an ischemic etiology, a NYHA class III dyspnea and reduced ejection fraction. Concerning the echocardiographic results, patients in AHF group had a more reduced ejection fraction compared to controls, a more dilated left ventricle and left atrium and pulmonary hypertension.

NT-proBNP has become the cornerstone biomarker for diagnosis and prognosis of AHF, being part of the guideline recommendations [2,62,63,64]. Our study confirmed the value of NT-proBNP in differentiating cases of acute decompensated HF, as the level was significantly higher in this group (AHF) compared to controls. The effects of NPs are complex, as they act on multiple physiological pathways. In addition to controlling natriuresis, diuresis and blood pressure, NP can influence ion channels and fibroblasts in cardiomyocytes, being potent and critical regulators of heart rate and arrhythmias [65]. Jeremy Springer et al. showed in their study that NPs can increase heart rate and electrical conduction by activating the guanylyl-cyclase-linked NPR-A and NPR-B receptors and inhibiting PDE3 activity. They observed reductions in the R–R interval, P wave duration and P–R interval on ECGs, correlated with BNP and CNP levels and increased spontaneous action potential frequency in isolated SAN myocytes, by increasing L-type Ca(2+) current (I(Ca, L)) and the hyperpolarization-activated current (I(f)) [66]. NPs have a particular relationship with atrial fibrillation (AF). AF is considered an independent predictor of increased BNP and NT-proBNP in dyspneic, mildly symptomatic or asymptomatic patients, given the elevated atrial pressures [67]. Furthermore, as previous studies showed, the NT-proBNP level was able to predict the recurrence of AF in mild HF and in a population-based cohort, independently from other cardiac comorbidities and diseases [67,68].

hs-cTnI had significantly higher values in the AHF group compared to controls in our study. In acute heart failure, the primary role of troponin measurement is to detect acute coronary syndrome as a precipitant of acute HF [69,70]. However, data from the PRIDE trial and the ADHERE Registry showed cardiac troponin concentrations above the detection limit in patients with acute HF, even when an acute coronary syndrome was excluded [71,72]. Troponin elevation in the setting of acute HF is considered due to cardiomyocyte injury and cell death. The pathophysiological mechanisms proposed include neurohormonal activation, realease of inflammatory cytokines, increased wall stress, oxidative stress and supply demand mismatch with subendocardial ischemia [73]. All of these mechanisms are decisive processes, influencing the prognosis. From this perspective, measurement of the troponin level brings additional prognostic information in patients with acute HF, as several studies confirmed [72,74,75]. Cardiac troponin was associated with readmission rates and mortality risk in patients with acute HF [69,75,76].

Furthermore, the complexity of mechanisms involved in the development of HF can be better explored by a multi-marker strategy. While elevated NP levels reflect myocardial stretch, elevated circulating troponin level is a marker of myocardial injury, bringing added value in prognostic assessment. Studies have confirmed this relationship, concluding that the combined measurement of high-sensitivity cardiac troponins and natriuretic peptides shows additional prognostic value and superior risk stratification in acute HF [77,78,79].

ECG is one of the first investigations indicated in patients with acute HF, being able to detect possible causes of decompensation, complications or even structural changes. As other studies have shown, the information provided by the ECG increases the diagnostic sensitivity for acute HF, since most cases of decompensated HF associate some ECG abnormalities [7,80]. Our data confirmed the value of ECG in the diagnosis of AHF, showing that the majority of patients with acute HF had ECG changes (81.6%), and compared to controls, the percentages were significantly higher. These results suggest that a normal ECG, in a clinical context similar to AHF, may support an alternative diagnosis.

Besides its value in establishing the diagnosis, ECG proved to have an important role in prognosis assessment, being able to predict in-hospital and long-term mortality or readmission risk [81,82]. In previous studies, abnormalities associated with poor prognosis included: presence of atrial fibrillation, presence of bundle branch block, prolonged corrected QT interval, prolonged QRS, presence of ST elevation or Q waves, higher heart rate [81,82,83]. For our patients with acute HF, the ECG more frequently showed a non-sinus rhythm, apparent U wave, PRWP and prolonged corrected QT interval, similar to other studies.

Holter ECG monitoring is not currently considered a basic diagnostic method for AHF but should not be excluded as an important tool in risk stratification. Early activation of neurohormonal system and influence of autonomic nervous system on cardiovascular system can lead to dynamic changes in electrocardiographic parameters, which can only be detected through continuous monitoring [8]. Moreover, Holter ECG allows the analysis of the heart electrical activity during the patients’ daily activities, which increases its predictive power. Holter parameters that have predictive value include: heart-rate variability, heart-rate turbulence, repolarization dynamics and variability, presence of arrhythmias, all of which play a role in predicting mortality and hospitalization [8,84,85]. Given the impact of these parameters on prognosis, we investigated whether their value is associated with AHF and the usual cardiac biomarkers. In our study, all heart-rate variability parameters (SDNN, SDANN, RMDDS, HRVTi) had similar values for AHF and controls, perhaps due to the fact that there were also patients with chronic HF in the control group. Heart-rate variability parameters also showed no correlations with common cardiac biomarkers in patients with AHF. Spot UNa was the only biomarker that showed correlation with SDNN, which supports its superior value in predicting adverse events. Since heart-rate variability parameters were not significantly influenced by the acute state, we conclude that the use of those parameters in AHF does not bring additional information.

Arrhythmias and HF have a close and bidirectional relationship. In HF, fibrosis, increased intracardiac pressure and ischemia predispose the vulnerable myocardial tissue to arrhythmias. At the same time, the presence of arrhythmias can precipitate acute decompensation of HF and favor the progressive deterioration of cardiac function, through arrhythmia-induced cardiomyopathy [86,87]. One of the most common heart rhythm disorders in HF is AF, given the multiple shared risk factors and mechanisms [88]. Adverse hemodynamic effects secondary to loss of atrial contraction and irregularity of ventricular contractions, intracardiac stasis and risk of embolism, often lead to unfavorable prognosis [86].

In this context, we expected to find an association between AHF and rhythm disturbances. On ECG, and also on Holter monitoring, atrial fibrillation was significantly associated with cases of AHF. However, when we looked for correlations with NT-proBNP, hs-cTnI or CK-MB, we did not find a relationship between biomarker levels and the presence of atrial fibrillation in our study, which means that they fail to predict supraventricular arrhythmias in patients with AHF. The only association we found was with spot urinary sodium, with lower values in the presence of atrial fibrillation on Holter monitoring. The inability to efficiently excrete sodium in patients with AHF is due to sustained activation of the renin–angiotensin–aldosterone system and sympathetic nervous system, which can precipitate paroxysmal arrhythmic events. Our results support the value of spot urinary sodium determination in patients with AHF as a predictor of atrial fibrillation.

Regarding ventricular arrhythmias detected by Holter monitoring, we found correlations between NT-proBNP and complex PVCs or ventricular tachycardia in patients with AHF, which shows that NT-proBNP levels may be an indicator of malignant ventricular arrhythmias. Complex PVCs were also correlated with troponin values in patients with AHF, which highlights its prognostic role. Ventricular arrhythmias are associated with an increased risk of sudden cardiac death in HF patients, as Chen et al. demonstrated in an analysis [89]. From this perspective, Holter monitoring may be the only investigation that can diagnose ventricular arrhythmias, and, given the strong association with NT-proBNP and hs-cTnI, its indication may be guided by cardiac biomarker levels.

Although we did not identified correlations of spot UNa with PVCs or ventricular tachycardia events, the presence of fragmented QRS (fQRS) was significantly associated with spot UNa. fQRS is an ECG abnormality associated with myocardial scarring and fibrosis. Consequently, its presence has been correlated in previous studies with a higher risk of ventricular arrhythmias, sudden cardiac death and all-cause mortality [11,90,91,92,93,94]. The relationship between spot UNa and fQRS highlights the value of spot UNa as a prognostic biomarker that can predict the risk of ventricular arrhythmias. Further studies are needed to confirm the hypothesis.

cQTi prolongation is an ECG parameter used frequently in clinical practice to assess the risk for malignant ventricular arrhythmias. As several studies have shown, the QT interval is associated with torsade de pointes and mortality [11,95,96,97]. In our study, cQTi had significantly higher values in patients with AHF, which confirms the impact that acute cardiac decompensation may have on electrical disturbances. NT-proBNP, and urinary ACR were correlated with higher cQTi values in patients with AHF, supporting their role in predicting electrical abnormalities.

A pathological Q wave is typically associated with prior myocardial infarction [98,99,100]. In heart failure, its role is related to the ischemic etiology, contributing at the same time to the prediction of adverse events. Ari Pelli et al. concluded in a prospective multicenter study that pathological Q waves are strong predictors of implantable cardiac defibrillator [101]. Furthermore, a study conducted on a general population showed that, even for patients without HF or ischemic heart disease, the presence of pathological Q wave is associated with poor prognosis [102]. Our study showed that the presence of pathological Q wave is correlated with NT-proBNP levels, which further confirms that this ECG abnormality may be a marker of HF decompensation.

Another ECG finding present in AHF group was a poor R-wave progression (PRWP), identified significantly more frequently than in controls. PRWP is associated, as Schröder et al. demonstrated, with adverse prognosis in general population and with sudden cardiac death in patients with coronary artery disease [103]. The prognostic role is supported also by Anttila et al., in an article published in the *European Heart Journal*, who concluded that PRWP independently predicted all-cause and cardiovascular mortality [104]. Regarding the correlation of PRWP with cardiac biomarkers, our study did not show a significant association.

ST-segment modifications were not significantly more frequent in patients with AHF in our study, but they presented correlations with troponin and CK-MB. Since both troponin and CK-MB are biomarkers associated with coronary syndrome, the correlation with ST-segment modifications was expected, as previously confirmed by several studies [105,106,107,108,109].

### Limitations of the Study

The present study has some limitations. The main limitation is the bias due to the single-center design and the small number of patients and controls enrolled in the study. In addition, only in-hospital adverse cardiovascular events were analyzed, which limited the possibility of evaluating the long-term prognosis. Data on long-term cardiovascular events and follow-up are planned to be included in a future study. We plan to expand our study to a larger number of cases that would allow the multivariable regression analysis of the data and the design of a multiparameter risk stratification score.

## 5. Conclusions

The objective of the present study was to assess the relationship between the usual cardiac biomarkers (NT-proBNP, hs-cTnI, CK-MB) and patterns of ECG or Holter ECG monitoring in patients with acute heart failure, in an adult population from Romania.

Patients with AHF had significantly more frequent abnormal ECG patterns, i.e., non-sinus rhythm, prominent U wave, prolonged cQT interval, PRWP and atrial fibrillation. Holter ECG parameters of heart rate variability were similar for the two groups. NT-proBNP correlated with QTi and cQTi, pathological Q wave, complex PVCs and ventricular tachycardia. hs-cTnI and CK-MB were associated with ST-segment modifications, and hs-cTnI alone with complex PVCs. We identified statistical significance for spot UNa and urinary ACR levels in AHF patients compared to the control group. They were also associated with fQRS, atrial fibrillation, QTi, cQTi and SDNN.

The statistical relationships found between cardiac biomarkers and ECG patterns support the added value of ECG in the diagnosis of AHF. We emphasize the importance of the proper ECG analysis of more subtle parameters that can be easily missed. As a non-invasive technique, ECG can be used in the outpatient setting as a warning signal announcing the acute decompensation of HF. In addition, the information provided by the ECG complements the biomarker results, supporting the diagnosis of AHF in cases of dyspnea of uncertain etiology. Further studies are needed to confirm long-term prognosis in a multi-marker approach.

## Figures and Tables

**Table 1:** Baseline characteristics of patients with acute HF and control group.

Characteristics	Total (*n* = 80)	Acute HF (*n* = 49)	Control Group (*n* = 31)	*p*-Value
Age, years	65.18 ± 12.98 (35–88)	67.98 ± 12.77 (35–88)	60.74 ± 12.22 (36–87)	0.014
Area of residence Urban, *n* (%) Rural, *n* (%)	40 (50%) 40 (50%)	25 (51.0%) 24 (49.0%)	15 (48.4%) 16 (51.6%)	0.818
Gender Male, *n* (%) Female, *n* (%)	51 (63.8%) 29 (36.3%)	28 (57.1%) 21 (42.9%)	23 (74.2%) 8 (25.8%)	0.122
Current smoker status, *n* (%)	39 (48.8%)	22 (44.9%)	17 (54.8%)	0.386
Excessive Alcohol consumption, *n* (%)	23 (28.8%)	13 (26.5%)	10 (32.3%)	0.581
Hypertension, *n* (%)	55 (68.8%)	37 (75.5%)	18 (58.1%)	0.101
Hyperlipidemia, *n* (%)	33 (41.3%)	23 (46.9%)	10 (32.3%)	0.194
Diabetes, *n* (%)	25 (31.3%)	17 (34.7%)	8 (25.8%)	0.403
Hyperuricemia, *n* (%)	39 (48.8%)	26 (53.1%)	13 (41.9%)	0.332
Obesity, *n* (%)	37 (46.3%)	22 (44.9%)	15 (48.4%)	0.760
CKD, *n* (%)	62 (78.5%)	42 (85.7%)	20 (66.7%)	0.046
Anemia, *n* (%)	22 (27.5%)	16 (32.7%)	6 (19.4%)	0.194
Infection, *n* (%)	20 (25.0%)	15 (30.6%)	5 (16.1%)	0.145
Atrial fibrillation, *n* (%)	34 (42.5%)	26 (53.1%)	8 (25.8%)	0.016
COPD, *n* (%)	14 (17.5%)	12 (24.5%)	2 (6.5%)	0.039
Ischemic heart disease, *n* (%)	29 (36.3%)	19 (38.8%)	10 (32.3%)	0.555
Infection, *n* (%)	20 (25.0%)	15 (30.6%)	5 (16.1%)	0.145
Mortality rate, *n* (%)	5 (6.3%)	5 (10.2%)	0 (0%)	0.066
Impaired self-care capacity, *n* (%)	18 (22.5%)	16 (32.7%)	2 (6.5%)	0.006

Abbreviations: HF = heart failure; CKD = chronic kidney disease; COPD = chronic obstructive pulmonary disease; *n* = number of subjects; % = percentage of subjects.

**Table 2:** Differences in therapy in AHF patients and control group.

Treatment	Total (*n* = 80)	Acute HF (*n* = 49)	Control Group (*n* = 31)	*p*-Value
Loop-diuretics, *n* (%)	62 (77.5%)	47 (95.9%)	15 (48.4%)	<0.001
MRA, *n* (%)	51 (63.8%)	35 (71.4%)	16 (51.6%)	0.072
ACEI/ARBs/ARNi, *n* (%)	48 (60.0%)	29 (59.2%)	19 (61.3%)	0.851
SGLT2i, *n* (%)	10 (12.5%)	8 (16.3%)	2 (6.5%)	0.193
Beta-blockers, *n* (%)	54 (67.5%)	33 (67.3%)	21 (67.7%)	0.971
Calcium-channel blockers, *n* (%)	18 (22.5%)	12 (24.5%)	6 (19.4%)	0.592
Digoxin, *n* (%)	7 (8.8%)	4 (8.2%)	3 (9.7%)	0.815
Amiodarone, *n* (%)	19 (23.8%)	16 (32.7%)	3 (9.7%)	0.019
Dobutamine, *n* (%)	6 (7.5%)	5 (10.2%)	1 (3.2%)	0.248
Vasopressor drugs, *n* (%)	4 (5.0%)	4 (8.2%)	0 (0.0%)	0.103
Nitroglycerine, *n* (%)	17 (21.3%)	17 (34.7%)	0 (0.0%)	0.001
Statine, *n* (%)	49 (61.3%)	33 (67.3%)	16 (51.6%)	0.159
Trimetazidine, *n* (%)	6 (7.5%)	4 (8.2%)	2 (6.5%)	0.777
CPAP non-invasive ventilation, *n* (%)	13 (16.3%)	13 (26.5%)	0 (0.0%)	0.002
Invasive ventilation, *n* (%)	4 (5.0%)	4 (8.2%)	0 (0.0%)	0.103

Abbreviations: HF = heart failure; MRA = mineralocorticoid receptor antagonists; ACEI = angiotensin-converting enzyme inhibitors; ARBs = angiotensin receptor blockers; ARNi = angiotensin receptor/neprilysin inhibitor; SGLT2i = sodium-glucose cotransporter-2 inhibitors; CPAP = continuous positive airway pressure; *n* = number of subjects; % = percentage of subjects.

**Table 3:** Laboratory findings in AHF and control group.

Laboratory Test	Total (*n* = 80)		Acute HF (*n* = 49)		Control Group (*n* = 31)		*p*-Value
	Mean ± STD	Range (Min–Max)	Mean ± STD	Range (Min–Max)	Mean ± STD	Range (Min–Max)	
Hemoglobin (g/dL)	13.28 ± 1.98	7.6–17.1	12.98 ± 2.18	7.6–17.1	13.76 ± 1.51	10.7–16.2	0.089
Hematocrit (%)	40.16 ± 5.97	24.5–55.9	39.81 ± 6.83	24.5–55.9	40.72 ± 4.33	32.7–48.5	0.509
Platelets (×10^3^/µL)	232.45 ± 73.64	59–411	234.63 ± 81.033	59–401	229 ± 61.27	130–411	0.741
Leukocytes (×10^9^/L)	10.34 ± 3.87	1.38–25.47	11.12 ± 4.32	1.38–25.47	9.11 ± 2.62	5.06–16.99	0.011
RBCs (×10^12^/L)	4.5 ± 0.73	2.7–6.5	4.48 ± 0.85	2.7–6.5	4.55 ± 0.49	3.5–5.4	0.667
CRP (mg/dL)	2.66 ± 3.98	0.03–24.50	2.52 ± 2.86	0.06–13.63	2.88 ± 5.34	0.03–24.50	0.690
Na^+^ (mmol/L)	138.44 ± 4.73	116–147	137.8 ± 5.62	116–147	139.45 ± 2.61	133–145	0.079
K^+^ (mmol/L)	4.49 ± 0.63	3.3–7.2	4.56 ± 0.719	3.3–7.2	4.37 ± 0.45	3.8–5.5	0.178
Cl^-^ (mmol/L)	101.57 ± 9.54	36–115	100.82 ± 11.44	36–115	102.92 ± 4.34	94–110	0.373
Ca^2+^ (mmol/L)	8.99 ± 0.58	7.68–10.70	8.87 ± 0.61	7.68–10.2	9.19 ± 0.46	8.44–10.7	0.016
Mg^+^ (mmol/L)	8.99 ± 0.58	1.46–3.11	2.06 ± 0.33	1.46–3.11	1.97 ± 0.16	1.58–2.25	0.118
Urea (mg/dL)	60.34 ± 31.92	17–184	69.69 ± 34.70	24–184	45.5 ± 19.71	17–95	0.001
Creatinine (mg/dL)	1.19 ± 0.49	0.53–3.54	1.32 ± 0.54	0.71–3.54	0.98 ± 0.30	0.53–1.81	0.002
Urinary ACR (mg/g)	152.38 ± 337.57	3–2256	231.61 ± 413.00	5–225	27.13 ± 26.48	3–122.3	0.007
Spot UNa (mEq/L)	71.61 ± 46.95	15–220	59.29 ± 34.58	15–128	91.10 ± 57.03	15–220	0.003
MAU (mg/L)	122.19 ± 252.67	4–1431	175.06 ± 308.189	4–143	38.63 ± 66.122	4–286	0.018
Spot urine creatinine (mg/dL)	104.84 ± 90.03	11–547.17	87.20 ± 65.04	11–258	132.72 ± 115.17	19.66–547.17	0.027
Uric acid (mg/dL)	7.30 ± 2.42	1.7–15.7	7.79 ± 2.49	1.7–15.7	6.47 ± 2.11	3.3–10.6	0.022
TSH (µIU/L)	2.58 ± 4.40	0.12–38	3.04 ± 5.57	0.14–38	1.88 ± 1.13	0.12–5.11	0.258
Feritin (µg/L)	220.13 ± 273.24	29–2100	232 ± 318.12	29–210	197.37 ± 158.61	36–560	0.618
Serum iron (µg/dL)	57.63 ± 33.44	11–233	50.10 ± 23.93	11–103	69.93 ± 42.51	25–233	0.024
Glucose (mg/dL)	149.21 ± 80.62	25–403	161.45 ± 86.78	25–403	127.79 ± 64.50	73–366	0.057
T-Col (mg/dL)	163.91 ± 50.74	79–292	162.14 ± 56.63	79–292	166.80 ± 40.05	101–238	0.695
LDL-c (mg/dL)	113.18 ± 48.23	34–247	116.13 ± 55.14	34–247	108.20 ± 33.81	45.8–172	0.433
HDL-c (mg/dL)	38.43 ± 13.74	11–75	36.49 ± 12.57	11–68	41.60 ± 15.16	18–75	0.109
TG (mg/dL)	114.96 ± 65.04	32–359	112.08 ± 59.94	36–354	119.67 ± 73.45	32–359	0.618
Bil (mg/dL)	1.08 ± 0.91	0.2–5.2	1.24 ± 1.08	0.2–5.2	0.83 ± 0.45	0.2–2.1	0.024
Lactic acid (mg/dL)	2.15 ± 2.06	0.50–10.80	2.28 ± 2.18	0.70–10.8	1.84 ± 1.77	0.50–7.40	0.495
Serum bicarbonate (mEq/L)	24.36 ± 4.95	15.90–38.30	23.98 ± 5.42	15.9–38.3	25.01 ± 4.01	19.20–34.80	0.420
GGT (U/L)	98.30 ± 115.77	11–804	121.22 ± 136.63	11–804	60.87 ± 53.02	13–216	0.007
ALP (U/L)	108.63 ± 52.19	31–348	123.57 ± 57.62	44–348	85.14 ± 30.64	31–168	<0.001
LDH (U/L)	263.56 ± 107.30	138–849	298.90 ± 120.25	149–849	207.03 ± 42.02	138–305	<0.001
Serum total proteins (g/dL)	6.71 ± 0.69	5–8.4	6.5 ± 0.71	5–8	6.93 ± 0.60	5.6–8.4	0.022
CK-MB (U/L)	41.16 ± 84.02	1.5–732	51 ± 105	1.5–732	26 ± 22	5.6–130	0.213
CK (U/L)	347.20 ± 905.00	1.5–4567	409 ± 965	12–603	251 ± 809	1.5–4567	0.453
Myoglobin (ng/mL)	375.13 ± 661.64	25.9–3500	290 ± 311	25.9–143	598 ± 1181	53–3500	0.489
D-dimer (ng/mL)	2.43 ± 2.14	0.04–8	2.57 ± 2.06	0.1–8	2.21 ± 2.26	0.04–7	0.470

Abbreviations: HF = heart failure; RBCs = red blood cells; CRP = C reactive protein; ACR = albumin to creatinine ratio; UNa = urinary sodium; MAU = microalbuminuria; TSH = thyroid stimulating hormone; T-Col = total cholesterol; LDL-c = low-density lipoprotein cholesterol; HDL-c = high-density lipoprotein cholesterol; TG = triglycerides; Bil = bilirubin; GGT = gamma-glutamyl transferase; ALP = alkaline phosphatase; LDH = lactate dehydrogenase; CK-MB = creatine kinase-MB; CK = creatine kinase; STD = standard deviation. Min = minimum value; Max = maximum value.

**Table 4:** Characteristics of acute HF patients.

Characteristics	Acute HF (*n* = 49)
Etiology of HF	
Ischemic, *n* (%)	24 (51.1%)
Alcoholic CMP, *n* (%)	5 (10.6%)
Valvular, *n* (%)	6 (12.8%)
HTN, *n* (%)	7 (14.9%)
NYHA class	
Class I, *n* (%)	1 (2.1%)
Class II, *n* (%)	11 (22.9%)
Class III, *n* (%)	28 (58.3%)
Class IV, *n* (%)	8 (16.7%)
Type of HF	
Reduced EF, *n* (%)	39 (81.3%)
Mildly reduced EF, *n* (%)	0 (0.0%)
Preserved EF, *n* (%)	9 (18.8%)

Abbreviations: HF = heart failure; HTN = hypertension; NYHA = New York Heart Association; EF = ejection fraction; CMP = cardiomyopathy; *n* = number of subjects; % = percentage of subjects.

**Table 5:** Echocardiography parameters in acute HF and control group.

Parameter	Total (*n* = 80)		Acute HF (*n* = 49)		Control Group (*n* = 31)		*p*-Value
	Mean ± STD	Range (Min–Max)	Mean ± STD	Range (Min–Max)	Mean ± STD	Range (Min–Max)	
LVEF (%)	35.02 ± 17.62	10–76	30.00 ± 15.87	10–65	42.96 ± 17.56	12–76	0.01
LA area (mm^2^)	27.67 ± 10.59	13.8–94	30.14 ± 12.16	13.8–94	23.78 ± 5.75	14.2–35.1	0.008
LVEDD (mm)	56.95 ± 8.47	38–74	58.28 ± 7.87	44–74	54.19 ± 8.19	38–74	0.029
RVEDD (mm)	35.37 ± 7.011	24–55	36.25 ± 7.04	25–55	34.00 ± 6.84	24–51	0.165
sPAP (mmHg)	38.94 ± 22.71	10–97	45.14 ± 22.51	10–97	29.31 ± 19.64	10–75	0.002
TAPSE (mm)	18.01 ± 4.09	7–26	17.59 ± 4.27	7–26	18.68 ± 3.77	11–25	0.251
MAPSE (mm)	12.03 ± 2.90	5–19	11.55 ± 2.70	7–19	12.81 ± 3.08	5–18	0.058

Abbreviations: HF = heart failure; LVEF = left ventricular ejection fraction; LA = left atrium; LVEDD = left ventricular end-diastolic diameter; RVEDD = right ventricular end-diastolic diameter; sPAP = systolic pulmonary artery pressure; TAPSE = tricuspid annular plane systolic excursion; MAPSE = mitral annular plane systolic excursion; Min = minimum value; Max = maximum value; STD = standard deviation.

**Table 6:** Cardiac biomarkers profile in acute HF and control group.

**Biomarker**	**Descriptives**		**Acute HF** **(*n* = 49)**		**Control Group** **(*n* = 31)**		***p*-Value** **(Mann–Whitney Test)**
			**Values**	**SEM**	**Values**	**SEM**	<0.001
**NT-proBNP** **(ng/L)**	Mean		11,361.57	1273.014	370.44	86.615	
	95% CI for mean	Lower bound	8802.00		193.54		
		Upper bound	13,921.14		547.33		
	5% trimmed mean		10,890.67		297.41		
	Median		7922.00		267.00		
	Variance		79,407,713.292		232,567.384		
	STD		8911.101		482.252		
	Minimum		1168		32		
	Maximum		30,000		2364		
	Range		28,832		2332		
	Interquartile range		13,187		394		
	Skewness		0.781	0.340	2.832	0.421	
	Kurtosis		−0.556	0.668	9.616	0.821	
	Shapiro–Wilk test		<0.001		<0.001		
**Biomarker**	**Descriptives**		**Acute HF** **(*n* = 49)**		**Control Group** **(*n* = 31)**		***p*-Value** **(Mann-Whitney Test)**
			**Values**	**SEM**	**Values**	**SEM**	<0.001
**hs-cTnI** **(ng/L)**	Mean		2688.029	1089.6966	558.547	342.4639	
	95% CI for mean	Lower bound	497.047		−140.857		
		Upper bound	4879.011		1257.952		
	5% trimmed mean		1256.013		184.500		
	Median		53.000		6.430		
	Variance		58,184,499.698		3,635,726.446		
	STD		7627.8765		1906.7581		
	Minimum		0.0		0.1		
	Maximum		36,701.0		9925.0		
	Range		36,701.0		9924.9		
	Interquartile range		349.0		19.5		
	Skewness		3.363	0.340	4.430	0.421	
	Kurtosis		11.124	0.668	20.867	0.821	
	Shapiro–Wilk test		<0.001		<0.001		

Abbreviations: HF = heart failure; NT-proBNP = N-terminal (NT)-pro B-type natriuretic peptide; hs-cTnI = high-sensitive cardiac troponin I; CI = confidence interval; STD = standard deviation; SEM = standard error of the mean.

**Table 7:** Log-transformed cardiac biomarkers profile in acute HF and control group.

**Biomarker**	**Descriptives**		**Acute HF** **(*n* = 49)**		**Control Group** **(*n* = 31)**		***p*-Value** **(Mann-Whitney Test)**
			**Values**	**SEM**	**Values**	**SEM**	<0.001
**log** **NT-proBNP** **(ng/L)**	Mean		3.8924	0.05928	2.2943	0.09041	
	95% CI for mean	Lower bound	3.7733		2.1097		
		Upper bound	4.0116		2.4790		
	5% trimmed mean		3.9034		2.2810		
	Median		3.8988		2.4265		
	Variance		0.172		0.253		
	STD		0.41494		0.50340		
	Minimum		3.07		1.51		
	Maximum		4.48		3.37		
	Range		1.41		1.87		
	Interquartile range		0.61		0.83		
	Skewness		−0.408	0.340	0.173	0.421	
	Kurtosis		−0.836	0.668	−0.919	0.821	
	Shapiro–Wilk test		0.018		0.148		
**Biomarker**	**Descriptives**		**Acute HF** **(*n* = 49)**		**Control Group** **(*n* = 31)**		***p*-Value** **(Mann-Whitney Test)**
			**Values**	**SEM**	**Values**	**SEM**	<0.001
**log** **hs-cTnI** **(ng/L)**	Mean		1.8838	0.19265	1.0818	0.20568	
	95% CI for mean	Lower bound	1.4964		0.6617		
		Upper bound	2.2711		1.5018		
	5% trimmed mean		1.9211		1.0248		
	Median		1.7243		0.8082		
	Variance		1.819		1.311		
	STD		1.34853		1.14517		
	Minimum		−1.52		−1.02		
	Maximum		4.56		4.00		
	Range		6.09		5.01		
	Interquartile range		1.32		0.94		
	Skewness		−0.213	0.340	1.110	0.421	
	Kurtosis		0.841	0.668	0.908	0.821	
	Shapiro–Wilk test		0.022		0.002		

Abbreviations: HF = heart failure; log NT-proBNP = log-transformed N-terminal (NT)-pro B-type natriuretic peptide; log hs-cTnI = log-transformed high-sensitive cardiac troponin I; CI = confidence interval; STD = standard deviation; SEM = standard error of the mean.

**Table 8:** ECG and Holter parameters in AHF and control group.

**Parameter**	**Total** **(*n* = 80)**		**Acute HF** **(*n* = 49)**		**Control Group** **(*n* = 31)**		***p*-Value**
	**Mean ± STD**	**Range** **(Min–Max)**	**Mean ± STD**	**Range** **(Min–Max)**	**Mean ± STD**	**Range** **(Min–Max)**	
HR (b/min)	96.96 ± 29.32	38–174	101.29 ± 28.77	38–160	90.13 ± 29.34	46–174	0.098
QRSi (ms)	88.81 ± 32.20	40–180	91.94 ± 33.55	40–180	83.87 ± 29.79	40–160	0.278
PRi (ms)	163.19 ± 39.86	100–310	160.83 ± 50.210	100–310	165.54 ± 26.72	120–230	0.687
QTi (ms)	365.15 ± 65.59	220–560	368.37 ± 73.77	220–560	360.37 ± 50.76	280–520	0.584
cQTi (ms)	422.61 ± 66.25	275–685	432.49 ± 79.32	275–685	407 ± 32.88	342–476	0.050
Average HR—Holter (b/min)	78.18 ± 19.01	45–136	77.13 ± 17.06	45–131	80.09 ± 22.43	47–136	0.561
PVCs—Holter (*n*)	1591 ± 4231	0–2436	2119.79 ± 5131.21	0–24,364	718.95 ± 1491.45	0–5419	0.121
PVCs—Holter (%)	1.81 ± 4.56	0–26.15	0.81 ± 1.51	0–26.15	0.32 ± 0.41	0–5.85	0.086

Abbreviations: HF = heart failure; HR = heart rate; QRSi = QRS interval; PRi = PR interval; QTi = QT interval; cQTi = corrected QT interval; PVCs = premature ventricular contractions. Min = minimum value; Max = maximum value; STD = standard deviation; *n* = number of subjects; % = percentage of subjects; ms = milliseconds; b/min = beats per minute.

**Table 9:** Pathological patterns on ECG and Holter in AHF and control group.

Pattern	Total (*n* = 80)	Acute HF (*n*= 49)	Control Group (*n*= 31)	*p*-Value
Abnormal ECG, *n* (%)	58 (72.5%)	40 (81.6%)	18 (58.1%)	0.021
Non-sinus rhythm, *n* (%)	33 (41.3%)	25 (51.0%)	8 (25.8%)	0.026
LBBB, *n* (%)	9 (11.3%)	7 (14.3%)	2 (6.5%)	0.280
prominent U wave, *n* (%)	6 (7.5%)	6 (12.2%)	0 (0.0%)	0.043
pathological Q wave, *n* (%)	15 (18.8%)	12 (24.5%)	3 (9.7%)	0.098
negative T wave, *n* (%)	33 (41.3%)	19 (38.8%)	14 (45.2%)	0.572
PRWP, *n* (%)	40 (50%)	30 (61.2%)	10 (32.3%)	0.012
LQRSV, *n* (%)	5 (6.3%)	3 (6.1%)	2 (6.5%)	0.953
fQRS, *n* (%)	17 (21.3%)	13 (26.5%)	4 (12.9%)	0.147
ST-segment modification, *n* (%)	12 (15%)	8 (16.3%)	4 (12.9%)	0.676
Atrial fibrillation—Holter, *n* (%)	22 (36.1%)	18 (46.2%)	4 (18.2%)	0.029
complex PVC’s—Holter, *n* (%)	39 (62.9%)	25 (62.5%)	14 (63.6%)	0.929
Ventricular tachycardia—Holter, *n* (%)	17 (27.9%)	13 (33.3%)	4 (18.2%)	0.205

Abbreviations: HF = heart failure; ECG = electrocardiogram; LBBB = left bundle branch block; PRWP = poor R wave progression; LQRSV = low QRS voltage; fQRS = fragmented QRS; PVCs = premature ventricular contractions; *n* = number of subjects; % = percentage of subjects.

**Table 10:** Holter ECG monitoring: parameters of heart-rate variability in AHF and control group.

HR Variability Parameters	Total (*n* = 40)		Acute HF (*n* = 22)		Control Group (*n* = 18)		*p*-Value
	Mean ± STD	Range (Min–Max)	Mean ± STD	Range (Min–Max)	Mean ± STD	Range (Min–Max)	
SDNN (ms)	89.70 ± 40.43	25–226	85.73 ± 39.62	25–226	94.56 ± 42.01	49–191	0.499
SDANN (ms)	72.50 ± 33.41	17–169	65.73 ± 29.40	17–161	80.78 ± 36.89	42–169	0.159
RMDDS (ms)	41.65 ± 36	12–168	49.73 ± 41.98	12–168	31.78 ± 24.65	12–120	0.102
HRVTi (ms)	365 ± 161.56	100–810	341.36 ± 145.67	100–810	393.89 ± 179.05	210–810	0.313

Abbreviations: HF = heart failure; SDNN = standard deviation of the NN intervals; SDANN = standard deviation of the average normal to normal intervals; RMSSD = root mean square of the successive differences; HRVTi = heart rate variability triangular index; Min = minimum value; Max = maximum value; STD = standard deviation.

**Table 11:** Correlations of biomarkers with ECG and Holter parameters in patients with AHF.

Parameter	NT-proBNP	hs-cTnI	CK-MB	Urinary ACR	Spot UNa
QRSi	*p* = 0.733; r = −0.05	*p* = 0.853; r = −0.025	*p* = 0.672; r = −0.062	*p* = 0.324; r = −0.144	*p* = 0.138; r = −0.215
PRi	*p* = 0.090; r = −0.354	*p* = 0.358; r = 0.196	*p* = 0.559; r = 0.125	*p* = 0.341; r = −0.203	*p* = 0.484; r = 0.150
QTi	*p* = 0.039; r = 0.296	*p* = 0.885; r = −0.021	*p* = 0.912; r = −0.016	*p* = 0.010; r = 0.365	*p* = 0.593; r = −0.078
cQTi	*p* = 0.027; r = 0.317	*p* = 0.397; r = 0.124	*p* = 0.786; r = 0.040	*p* = 0.001; r = 0.445	*p* = 0.782; r = −0.040
Average HR—Holter	*p* = 0.411; r = 0.134	*p* = 0.939; r = 0.013	*p* = 0.553; r = 0.097	*p* = 0.553; r = −0.097	*p* = 0.152; r = −0.231
SDNN—Holter	*p* = 0.579; r = 0.125	*p* = 0.346; r = −0.211	*p* = 0.297; r = −0.233	*p* = 0.948; r = 0.015	*p* = 0.025; r = −0.476
SDANN—Holter	*p* = 0.746; r = 0.073	*p* = 0.458; r = −0.167	*p* = 0.346; r = −0.211	*p* = 0.906; r = −0.027	*p* = 0.075; r = −0.388;
RMDDS—Holter	*p* = 0.417; r = 0.182	*p* = 0.373; r = −0.200	*p* = 0.325; r = −0.220	*p* = 0.890; r = 0.031	*p* = 0.056; r = −0.414;
HRVTi—Holter	*p* = 0.960; r = 0.011	*p* = 0.453; r = −0.169	*p* = 0.304; r = −0.230	*p* = 0.773; r = 0.065	*p* = 0.095; r = −0.365
PVCs (%)—Holter	*p* = 0.165; r = 0.224	*p* = 0.722; r = −0.058	*p* = 0.449; r = −0.123	*p* = 0.353; r = 0.151	*p* = 0.713; r = −0.060

Abbreviations: QRSi = QRS interval; PRi = PR interval; QTi = QT interval; cQTi = corrected QT interval; HR = heart rate; SDNN = standard deviation of the NN intervals; SDANN = standard deviation of the average normal to normal intervals; RMSSD = root mean square of the successive differences; HRVTi = heart rate variability triangular index; NT-proBNP = N-terminal (NT)-pro B-type natriuretic peptide; hs-cTnI = high sensitive cardiac troponin I; CK-MB = creatine kinase-MB; ACR = albumin to creatinine ratio; UNa = urinary sodium.

**Table 12:** Correlations of biomarkers with ECG and Holter patterns in patients with AHF.

Pattern	NT-proBNP	hs-cTnI	CK-MB	Urinary ACR	Spot UNa
Pathological Q wave	*p* = 0.029; F = 5.057	*p* = 0.236; F = 1.440	*p* = 0.958; F = 0.003	*p* = 0.562; F = 341	*p* = 0.434; F = 0.624
Prominent U wave	*p* = 0.140; F = 2.251	*p* = 0.815; F = 0.055	*p* = 0.713; F = 0.137	*p* = 0.968; F = 0.002	*p* = 0.759; F = 0.095
Negative T wave	*p* = 0.672; F = 0.182	*p* = 0.265; F = 1.275	*p* = 0.867; F = 0.028	*p* = 0.359; F = 0.859	*p* = 0.723; F = 0.127
PRWP	*p* = 0.512; F = 0.437	*p* = 0.860; F = 0.031	*p* = 0.564; F = 0.337	*p* = 0.072; F = 3.384;	*p* = 0.916; F = 0.011
fQRS	*p* = 0.272; F = 1.236	*p* = 0.797; F = 0.067	*p* = 0.555; F = 0.353	*p* = 0.472; F = 0.527;	*p* = 0.020; F = 5.814
ST-segment modification	*p* = 0.215; F = 1.582	*p* = 0.038; F = 4.550	*p* = 0.018; F = 6.021	*p* = 0.720; F = 0.130	*p* = 0.221; F = 1.536
LBBB	*p* = 0.378; F = 0.792	*p* = 0.796; F = 0.068	*p* = 0.808; F = 0.060	*p* = 0.202; F = 1.671	*p* = 0.684; F = 0.168
Complex PVCs—Holter	*p* = 0.034; F = 4.857	*p* = 0.031; F = 4.987	*p* = 0.110; F = 2.678	*p* = 0.890; F = 0.019	*p* = 0.085; F = 3.121
Atrial fibrillation—Holter	*p* = 0.797; F = 0.067	*p* = 0.969; F = 0.002	*p* = 0.421; F = 0.663	*p* = 0.727; F = 0.124	*p* = 0.005; F = 8.968
Ventricular Tachycardia—Holter	*p* = 0.048; F = 4.167	*p* = 0.298; F = 1.115	*p* = 0.474; F = 0.524	*p* = 0.684; F = 0.168	*p* = 0.236; F = 1.451

Abbreviations: LBBB = left bundle branch block; PRWP = poor R wave progression; LQRSV = low QRS voltage; fQRS = fragmented QRS; PVCs = premature ventricular contractions; NT-proBNP = N-terminal (NT)-pro B-type natriuretic peptide; hs-cTnI = high-sensitive cardiac troponin I; CK-MB = creatine kinase-MB; ACR = albumin-to-creatinine ratio; UNa = urinary sodium.

## Data Availability

Data sharing not applicable.
